# Engineering soluble T‐cell receptors for therapy

**DOI:** 10.1111/febs.15780

**Published:** 2021-03-10

**Authors:** Ross A. Robinson, Catriona McMurran, Michelle L. McCully, David K. Cole

**Affiliations:** ^1^ Immunocore Ltd. Abingdon UK

**Keywords:** affinity‐enhanced; bispecific T‐cell engagers, cancer immunotherapy, peptide–human leukocyte antigen, T‐cell receptor

## Abstract

Immunotherapy approaches that target peptide–human leukocyte antigen (pHLA) complexes are becoming highly attractive because of their potential to access virtually all foreign and cellular proteins. For this reason, there has been considerable interest in the development of the natural ligand for pHLA, the T‐cell receptor (TCR), as a soluble drug to target disease‐associated pHLA presented at the cell surface. However, native TCR stability is suboptimal for soluble drug development, and natural TCRs generally have weak affinities for pHLAs, limiting their potential to reach efficacious receptor occupancy levels as soluble drugs. To overcome these limitations and make full use of the TCR as a soluble drug platform, several protein engineering solutions have been applied to TCRs to enhance both their stability and affinity, with a focus on retaining target specificity and selectivity. Here, we review these advances and look to the future for the next generation of soluble TCR‐based therapies that can target monomorphic HLA‐like proteins presenting both peptide and nonpeptide antigens.

## Graphical abstract

Native TCR stability and affinity are suboptimal for soluble drug development, limiting their potential as soluble drugs. To overcome these limitations and make full use of the TCR as a soluble drug platform, several protein engineering solutions have been applied to TCRs to enhance both their stability and affinity, with a focus on retaining target specificity and selectivity.

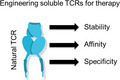

**Abbreviations**
5‐OP‐RU: 5‐(2‐oxopropylideneamino)‐6‐d‐ribitylaminouracil
CAR: chimeric antigen receptor
CD1a: CD1b, CD1c or CD1d, cluster of differentiation 1 a, b, c and d
CDR: complementarity‐determining region
HLAs: human leukocyte antigens
ImmTAC: Immune‐mobilising monoclonal TCRs Against Cancer
MR1: MHC‐related protein 1
pHLA: peptide–human leukocyte antigen
TCR: T‐cell receptor
TRuCs: T‐cell receptor fusion constructs

## Introduction

**Fig. 1:**
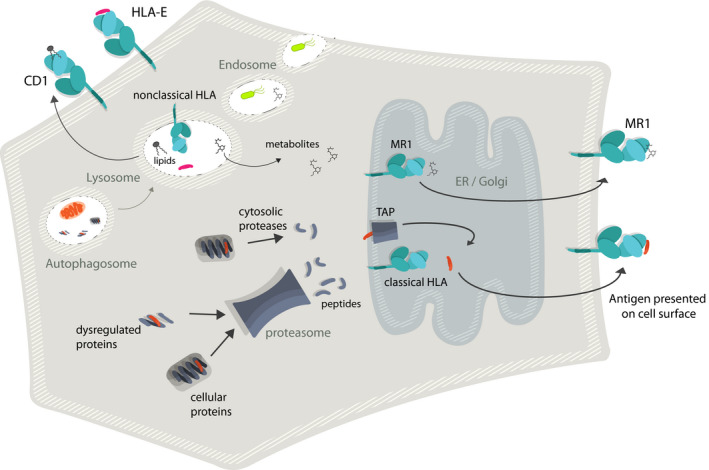
Presentation pathways of TCR ligands. Cartoon schematic showing the simplified antigen presentation pathways and cell surface expression of HLA class I (classical), HLA‐E, CD1 and MR1. HLA or HLA‐like molecules are coloured green and cyan with peptide ligands shown in red, metabolite ligands shown as black lines and lipid ligand depicted in black in cartoon representations of lipid tails and head groups

In recent years, there has been growing interest in human leukocyte antigens (HLAs) as targets for immunotherapy because they can present fragments of degraded proteins, representing virtually all intracellular and extracellular proteins, at the cell surface (Fig. 1). These pHLA complexes can, therefore, act as a ‘cellular window’ into both foreign protein expression during infection, protein dysregulation/mutation in cancer and recognition of self‐proteins in autoimmunity. However, targeting pHLA comes with some inherent protein engineering challenges. First, disease‐associated pHLA can be presented at very low levels on target cells (often below 10 copies of each specific peptide epitope per cell), representing a receptor occupancy challenge for any soluble targeting molecule [1]. Second, HLA is a self‐protein expressed by virtually all nucleated cells, so peptide‐dependent recognition is essential to avoid broad on‐target toxicity [2, 3]. Third, HLAs are highly polymorphic, which potentially limits patient coverage based on genetic background.

The natural pHLA ligand is the T‐cell receptor (TCR), a membrane‐spanning protein made up of two chains (α and β), each forming two domains (constant: C and variable: V). The TCR recognises a continuous pHLA surface consisting of two parallel alpha helices with a peptide bound in the cleft formed between them [4]. Structural studies have shown that the variable domains of both chains of the TCR directly interact with the exposed residues in the presented peptide and the surface of the HLA‐binding groove [5, 6]. These interactions orient the TCR diagonally over the pHLA surface with three complementarity‐determining region (CDR) loops, two germline‐encoded (CDR1 and CDR2) and one hypervariable (CDR3) per chain, mediating the interaction. Generally, interactions with the central solvent‐exposed regions of the peptide are dominated by the hypervariable CDR3 loops of the TCR, whilst additional interactions with the N and C peptide termini are often made by the CDR1α and CDR1β loops, respectively [7, 8]. In contrast, the CDR1 and CDR2 loops usually form the majority of contacts with the HLA helices, with different levels of contributions from the CDR3 loops normally governed by small differences in the positioning of the TCR and the lengths of the CDR3 loops [7, 8].

In this review, we will discuss some of the challenges associated with targeting pHLA using soluble TCRs and focus on solutions to engineer these receptors with the attributes required to generate an efficacious soluble therapeutic molecule. We consider approaches for optimising the stability/developability, affinity and specificity of TCRs for therapy. We have implemented many of these approaches practically in the generation of ImmTAC (Immune‐mobilising monoclonal TCRs Against Cancer) molecules, which comprise a monovalent affinity‐enhanced TCR (*K*_D_ ˜ pM range) fused to a monovalent anti‐CD3 single‐chain Fv antibody fragment [9, 10]. These soluble therapeutic bispecifics decorate tumour cells *via* the affinity‐enhanced TCR‐pHLA interaction, enabling redirected killing of tumour cells by T cells *via* CD3 engagement. Importantly, the very strong affinity of the engineered TCRs, combined with the potent triggering via the anti‐CD3 moiety, enables T‐cell redirection against tumour cells with low‐level presentation of tumour‐associated pHLAs [9]. Last, we consider the opportunities and protein engineering challenges associated with targeting monomorphic HLA‐like molecules with soluble TCRs, which have the potential to access different classes of antigen (including lipids and metabolites) and are unrestricted by genetic background.

### The natural poor stability and micromolar binding affinity of soluble TCRs are suboptimal for the development of soluble therapeutics

Native TCRs have been found to be relatively unstable as soluble molecules, likely reflecting their natural expression as transmembrane proteins at the cell surface. This characteristic has complicated the therapeutic development of these proteins as soluble reagents [11]. Additionally, although the buried surface area created at the TCR‐pHLA protein–protein interface is large (around 2000 Å^2^ on average), the naturally measured binding affinity is relatively weak (K_D_ ˜ 0.1 – 1000 µM) compared with other Ig‐like proteins [7, 12]. TCRs selective for tumour‐associated pHLAs tend to bind towards the weaker end of this range, likely representing the thymic deletion of TCRs binding with strong affinity to self‐derived tumour peptides, adding to the challenge of TCR selection and their utility as therapeutics in this disease area [12, 13, 14].

Although it is not fully understood why TCRs are naturally selected with these binding characteristics, it has been suggested that weak affinity might enable TCRs to cross‐react with many thousands, or even millions, of peptides in order to provide sufficient immune coverage against all possible foreign antigens [15, 16, 17, 18] and that the weak affinity is required for optimal T‐cell triggering [19, 20, 21]. In the case of bispecific T‐cell engagers, which typically use an anti‐CD3 antibody as the effector moiety, T‐cell triggering then becomes dependent on the binding affinity and kinetics of the anti‐CD3‐CD3 interaction. In theory, this interaction should enable serial triggering of the TCR‐CD3 complex on the redirected T cell (i.e. a weak enough affinity to allow serial engagement of multiple TCR‐CD3 complexes) and should fulfil the criteria of the kinetic proofreading model (i.e. the TCR must be engaged for a sufficient duration to allow signal initiation) [22]. It has also been observed that T cells transduced with a chimeric antigen receptor (CAR) of strong affinity for its ligand have a limited ability to penetrate solid tumours [23], suggesting that weak TCR affinity may enable T cells to disengage from neutralised targets, enabling the serial killing of other cells and increased penetration into tissues. Whatever the biological reason for the weak affinity of natural TCRs, the consequence for designing a TCR‐based soluble pHLA targeting bispecific is that, because of the very low natural presentation levels observed for many disease‐associated pHLA (often in the 10s of copies per cell) [1, 24], binding in the femto‐ to picomolar affinity range is needed to achieve a therapeutically relevant receptor occupancy level [9, 25].

With the inherent issues associated with natural TCRs, an obvious alternative to directly target pHLA as soluble therapeutics (given the ˜ 20 years R&D and proven stability and pharmacokinetic properties) is to use antibodies, which are naturally stable as soluble molecules and generally have stronger native affinity for their targets. However, despite a rich vein of academic literature [24, 38] and several commercial efforts, there are currently no clinical data available for soluble monoclonal antibody‐based bispecific molecules targeting pHLA (so‐called TCR‐mimic antibodies). One possible difficulty is the specificity of these reagents. Indeed, we recently demonstrated that some TCR‐mimic antibodies bind with a hotspot‐driven energetic binding mode, compared with the much broader binding mode typically employed by TCRs. Consequently, the TCR‐mimic antibodies engaged a minimal peptide motif, resulting in the recognition of a diverse peptide repertoire compared with the TCRs, leading to cross‐reactivity against target‐negative healthy cell lines [39]. These observations highlight the last issue to consider when engineering TCRs as soluble drugs: the TCR is thymically selected with binding characteristics that enable it to recognise foreign pHLA, whilst remaining tolerant to self pHLA. Even small modifications to the TCR outside of thymic selection can have unpredictable and unwanted consequences [40, 41, 42]. Therefore, it is vital to have a deep understanding of the parameters governing TCR‐pHLA interactions so that the interface can be modified in an intelligent manner that produces a molecule with favourable therapeutic characteristics (stable and of strong affinity), whilst remaining selective for the intended target.

## Engineering stable, soluble TCRs

As already discussed, its poor natural stability/solubility limits the scope to study the TCR as a soluble entity, with obvious knock‐on consequences for engineering a therapeutic soluble form of the molecule. This is reflected by relatively low expression yields, the relatively high levels of misfolding and aggregation observed for soluble TCRs, and the poor expression using protein display platforms, compared with antibodies. An additional confounding observation that has not been formally demonstrated, but is strongly hinted at by studies demonstrating preferential TCR expression at the cell surface [43], is that despite apparently unbiased pairing behaviour in the immune repertoire [44, 45], certain TCR Vα‐Vβ pairings seem to be more compatible as soluble molecules than others. Recent developments in single‐cell RNA sequencing are increasingly being applied to investigate the specificity of paired αβTCR sequences, representing a potential future resource to guide further engineering work by identifying TCR Vα‐Vβ pairings that are more likely to generate stable, specific, soluble reagents [46]. Historical, and more recent, approaches to address these issues and nuances are discussed below.

### Early attempts to generate soluble TCRs

**Fig. 2:**
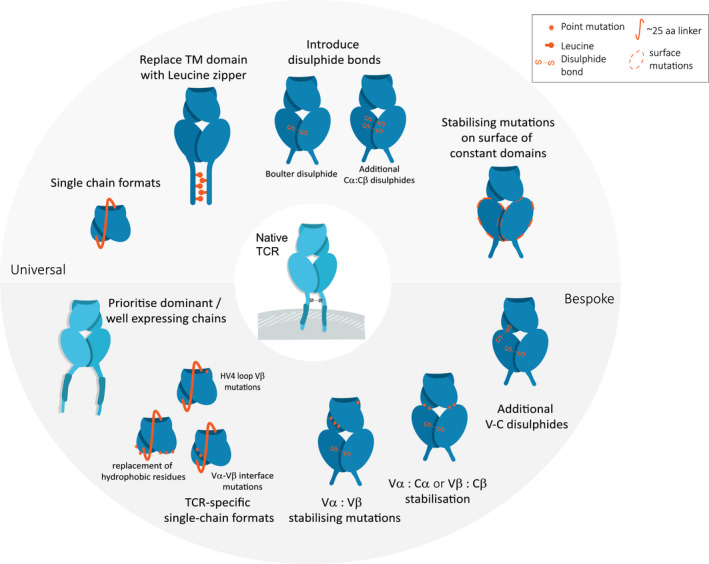
Protein engineering approaches to stabilise and solubilise TCR molecules. Cartoon schematics showing the different strategies that have been used to improve the stability and solubility of TCR molecules. TCR shown in blue, with modifications in orange. The types of modifications have been split into those that can be used universally (i.e. not dependent on the Vα‐Vβ pairing) and those which are bespoke (i.e. dependent on the Vα‐Vβ pairing).

Initial attempts to generate soluble TCRs followed the same strategies used to generate soluble single‐chain Fv antibodies, due to the large degree of sequence and structural similarity between the two molecules [47]. Several methods for linking the Vα and Vβ domains have been reported for both *E*. *coli* expression and yeast display, with a ˜ 25aa linker between the TCR Vβ C terminus and the TCR Vα N terminus being the most common approach (Fig. 2) [48]. However, the majority of scTCRs that contained just the Vβ domain linked to the Vα domain have been found to be poorly soluble with very low expression, likely due to the exposure of several surface hydrophobic residues normally buried by the C domains. Thus, computational modelling [47] and random mutagenesis *via* yeast display [49] or phage display [50] have been used to identify beneficial mutations, including the replacement of surface‐exposed hydrophobic residues primarily at the Vα‐Cα or Vβ‐Cβ interfaces, introduction of stabilising mutations at the Vα‐Vβ interface, mutations in the hypervariable 4 Vβ loop, or alterations that rigidify the linker sequence (Fig. 2). However, due to the high level of diversity of the Vα/Vβ germlines, these mutations are often unique solutions for individual TCRs, limiting the identification of universally beneficial mutations. As such, it is likely that for each scTCR generated, a structurally guided approach [47], and/or the use of random mutagenesis in a large display library [49], would be required to generate a stable soluble molecule.

### Universal stabilisation of the TCR C domain

Alternative approaches using the native four domain VαCα and VβCβ structures (thus bypassing the issue of exposing hydrophobic residues in the V domains) were later developed, providing more universal methods for stabilising soluble TCRs. This involved artificial improvements to aid the pairing of the extracellular α and β domains by either fusing an immunoglobulin kappa constant domains to the C terminus of both constant domains [51] or inserting *jun‐fos* leucine zippers at the TCR C terminus (Fig. 2). The leucine zipper method allowed expression of the TCR as a homogeneous product in insect cells, with a significant yield improvement compared with the expression without the engineered domains [52]. This leucine zipper strategy was later adapted for expression in *E. coli* as inclusion bodies, from which TCRs were successfully folded for biophysical analysis [53]. However, due to the length and flexibility of the leucine zipper, this method was less amenable to structural studies using X‐ray crystallography, or for therapeutic development. Thus, a novel interchain disulfide bridge (Boulter disulfide) between the two TCR C domains was developed [11], enabling relatively high‐yield production of stable, soluble protein from refolded inclusion bodies (Fig. 2). Because this method did not require the inclusion of any unstructured linkers, it was successfully adopted for higher throughput crystallisation screens [54] and for the creation of soluble TCR‐based bispecifics [55].

### Bespoke stabilisation of individual TCRs using computational and protein display methods

Although the approaches detailed above have generally enhanced our ability to generate soluble TCRs for study and therapeutic development, there remains substantial room for improvement through bespoke modifications to individual TCRs. For instance, the nature of the V domain pairing is known to influence presentation and surface stability of TCRs [43], suggesting that more tailored approaches could be of benefit to certain TCRs to fully optimise their stability for use as therapeutics. A recent study by Thomas et al [56] identified multiple dominant TCRs (including those encoding TRAV38‐1, TRAV38‐2, TRBV5‐1 and TRBV7‐8) that were significantly enriched at the cell surface when challenged with an introduced synthetic TCR, suggesting that these pairings occurred in a preferential manner. Although the effects on soluble TCR stability were not investigated, structural modelling and mutational screening were used to demonstrate that cell surface expression levels of poorly expressed TCRs could be rescued through the introduction of three residues present in the dominant TCR pairings: L96α (which resulted in a stabilisation at the Vα‐Cα interface) and R9β/Y10β (which resulted in a stabilisation at the Vβ‐Cβ interface). Additionally, a recent computational analysis of crystal structures and *in silico* modelling have been successful in improving both the yield and stability of soluble TCR heterodimers, even in the absence of the engineered Boulter disulfide [57]. The entire C domains were computationally screened to find positions where alternative residues were predicted to stabilise the TCR. Interestingly, many of the mutations selected were shown to locate to the solvent‐exposed surface and modify intrachain Cα‐Cα and Cβ‐Cβ, as well as interchain Cα‐Cβ interactions. Whereas individual mutations predicted to improve overall stability only resulted in small gains in resistance to thermal denaturation and mammalian expression titres, combining several mutations resulted in significant improvements, with the best full‐length TCR gaining 8.5 °C in melting point. Another study building on a scaffold already containing the Boulter disulfide algorithmically identified potential additional disulfide bonds that could be placed within the TCR C domains or at the V‐C interface based on existing crystal structure data [58]. Although the addition of these extra disulfide bonds showed only incremental improvements in thermal stability (+1‐3 °C determined using differential scanning calorimetry), the approach highlights the additional potential for fine‐tuning of certain Vα‐Vβ pairs.

Alongside such rational structure‐guided engineering, directed evolution has been highly fruitful in identifying stabilising mutations in TCRs. Error‐prone PCR of the framework region of the single‐chain murine 2C TCR was required to find mutations, which allowed reasonable surface display on yeast (correlating with the stability of the soluble molecule) [59], with mutations that increased surface display and thermostability predominantly occurring at the interface between Vα and Vβ regions. More sophisticated library designs that targeted this interface for a saturation mutagenesis approach [60] identified a single point mutation that enhanced both thermostability and binding affinity through an allosteric ‘long‐range’ driven mechanism at the interdomain interface, a phenomenon also seen in antibodies [61]. These findings demonstrate that TCR stability and TCR‐pHLA affinity are linked, which has important implications for designing protein engineering solutions to generate affinity‐enhanced TCRs. In another study, scTCR libraries with framework mutations were expressed by phage display and screened for improved thermostability. These investigations identified a dominant mutation in the murine 4B2A1 TCR Vβ (L214S) that replaced a hydrophobic surface‐exposed amino acid with a hydrophilic amino acid, which gave increased resistance to denaturation and improved soluble periplasmic expression yields [50].

### Summary

A common thread in recent TCR engineering publications is the integration of structurally guided information with large‐scale screening techniques to create a targeted library building approach.

The work of both Froning [57] and Harris [62] began with a systematic screen of individual point mutations to identify single residue changes that improved binding and/or stability. The use of a computational or physical deep mutational scan allowed the identification of key residues to prioritise in combinatorial screening. As discussed above, the most successful TCR stability and affinity engineering have resulted from TCR‐specific mutagenesis with limited universal applicability for this diverse molecule class. In the absence of any novel engineering strategies such as the Boulter disulfide, which can universally improve TCR stability/solubility, the future of TCR engineering may rely on optimising bespoke chain or chain‐pair specific scaffolds, requiring the greater use of *in silico* modelling and more sophisticated higher throughput screening tools. Additionally, advances in the field of TCR engineering, as well as more structural and experimental information, should enable the development of more sophisticated predictive tools for assessing stability and general developability of different TCR sequences, as has been the case for the antibody field [63, 64].

## Engineering TCR affinity

Several different strategies have been employed to enhance the naturally weak affinity of the TCR‐pHLA interaction. These methods fall broadly into two camps: 1) structurally guided *in silico* affinity maturation and 2) directed evolution using mammalian, yeast or phage display libraries (**Table** **1**). *In silico* approaches generally use molecular dynamics simulations of TCR‐pHLA complexes, including free energy calculations, to model mutations at the interface that might increase binding strength. Although *in silico* methods have been successful, they have generated relatively modest affinity increases (up to ˜ 400‐fold) [65, 66, 67, 68, 69], probably reflecting the highly complex and dynamic interface between the TCR and pHLA which is difficult to fully deconvolute using simulation alone. Indeed, molecular dynamics simulation analysis of affinity‐enhanced TCRs compared with their parental wild‐type TCRs highlighted that these changes in sequence do not necessarily add new contacts but have complementary, indirect or potentially stabilising effects [70].

**Table 1:** Overview comparing affinity enhancement display systems using phage (M13 filamentous), yeast (*Saccharomyces cerevisiae*) and mammalian cells. Neddylated substrates and E3 ligases responsible for the modification

Display system	Max. library size (# of cells that can be screened)	Genotype–phenotype link	Display of mTCR format	FACS sorting (for affinity and expression levels)	Refs
Phage (M13 filamentous)	10^12^ −10^13^	✓	✓	✕	[114]
Yeast (*Saccharomyces cerevisiae*)	10^9^ −10^10^	✓	✕ Only single‐chain formats	✓	[115, 116]
Mammalian	˜10^6^	✕ Not robust for transient transfection ✓ Standard viral transduction can result in heterogeneous expression due to varying sites of integration	✓	✓	[72, 117, 118]

Directed evolution uses large fully or semi‐randomised libraries of TCR displayed on mammalian cells, yeast or phage for affinity selection, usually using soluble target pHLA molecules. Typically, several rounds of selection are required to reach sequence convergence and selection of mutations for further analysis. Mammalian display methods for TCR affinity enhancement, using TCR‐negative T‐cell lines or other common mammalian cell lines, have been developed, allowing the use of cells derived from the same species throughout the affinity enhancement and protein production/scale‐up phases [71, 72]. However, due to cell culturing constraints, mammalian display methods using transient transfection have limited library sizes (˜10^5^–10^6^), possibly explaining the relatively modest affinity improvements that have been achieved so far (˜15‐fold). Additionally, the introduction of multiple transcripts per cell can result in the loss of genotype–phenotype link and the display of heterogeneous TCRs on the cell surface. Although recent developments in retroviral mammalian systems using CRISPR/Cas9 integration of TCR genes into a single genomic locus, enabling stable expression and transcriptional normalisation [73], overcome some of the issues of transient transfection [71, 72, 74, 75], nonmammalian display methods are currently better established for the generation of affinity‐enhanced TCRs.

Yeast display methods have shown particular promise with single‐chain formats that have been used to generate TCRs with low nanomolar affinities [59, 76, 77, 78], and have the key advantage that the stable display on the yeast surface tends to correlate with the stability of the soluble TCR. However, yeast display libraries are generally of medium size (˜10^9^–10^10^), limiting the diversity of the selection, and the display of heterodimeric VαCα/VβCβ TCRs has been problematic, likely due to misfolding events and the inherent instability of the TCR [49]. By contrast, phage display, with its large potential library size (˜10^12^–10^13^), stable genotype–phenotype link and cost‐effectiveness, is arguably the most successful method of generating clinically translatable soluble TCRs. Moreover, heterodimeric VαCα/VβCβ TCRs can be robustly expressed on phage particles. Phage display using a CDR walking library design, targeting up to six sequential amino acid positions at a time with degenerate NNK codons and combining the resulting mutations, has been successful in dramatically increasing the affinity of TCRs into the low picomolar–high femtomolar range [9, 25, 79]. However, phage display still has its limitations. For instance, the combinatorial effect of randomising multiple residues soon leads to unmanageably large libraries sizes (e.g. testing all alternative amino acid combinations at just 12 positions in a TCR would give a library size of more than 10^13^, already 100‐fold larger than the 10^11^ maximum practical phage library) [80]. This can be overcome by targeting only the central residues in the CDR loops, or the use of multiple separate libraries.

### Structurally guided protein display

As the number of residues that can be varied in a round of affinity enhancement is limited, tertiary complex structural information, which provides greater molecular detail of the TCR‐pHLA interface, could be used to select which residues to include in a library to enhance affinity or selectivity. For instance, libraries based on specific crystallographic data offer the potential to fix key residues at the TCR‐pHLA interface and allow flanking residues in the CDR loop to be randomised, as well as influencing the choice of encoding schemes for the selected TCR residues based on the chemical nature of the target site on the pHLA surface [72]. In addition, structurally guided libraries could also be used to maximise TCR interactions with multiple peptide side chains, as this binding mode has been associated with greater peptide selectivity [39]. Consequently, crystal structure data could provide the key information to design libraries that are more likely to increase interactions with underrepresented peptide residues (in terms of TCR contacts) to ‘distribute’ TCR binding energy more broadly over multiple peptide residues and enhance specificity [39]. Structural information also has the potential to identify amino acids that are proximal in the structure, but not necessarily in linear sequence. These residues can be combined in the same library to reduce mutation of residues that are not likely to yield affinity gains, allowing for beneficial covariance effects to come through in the resulting clones. A crystal structure may also reveal, due to the mode of binding, that a particular CDR loop does not contribute to the pHLA binding interface. Thus, structural guided approaches could be used to add residues to the loop and randomise using display methods, extending a particular loop to make new contacts, with the potential to increase affinity/specificity.

### Summary

As the field of TCR engineering matures, strategies that combine the best of both rational engineering and directed evolution provide a promising direction for future work. The use of a computational or physical deep mutational scans allows the identification of key residues to prioritise in combinatorial screening [81]. This could help to reduce the introduction of carrier mutations from multiple degenerate codons, which may either increase immunogenicity or introduce unfavourable characteristics without contributing to the favourable properties of a selected TCR. As the availability of structural information grows into a larger resource, we expect this integrated strategy to become the norm for TCR engineering.

## Engineering TCR specificity

A key consideration when affinity enhancing TCRs outside the rigours of thymic selection is the effect on TCR cross‐reactivity. However, it is important to separate the thresholds of cross‐reactivity when considering a TCR expressed on the surface of a T cell compared with a soluble bispecific TCR molecule. For the former, cross‐reactivity with nontarget antigens could occur if the binding affinity of the TCR is modified into the weak micromolar range for a particular self‐antigen (there have been multiple reports of functional TCR signalling with TCRs that bind with an affinity as low as K_D_ ˜ 1000 µM) [12, 82]. In contrast, for a soluble bispecific TCR, the threshold is likely to be in the low nM range (depending on the density of the target, the concentration of the soluble TCR and the effector function employed) in order to reach a functionally relevant receptor occupancy level on the surface of a target cell [9]. To put it another way, as long as there is an affinity window between a target antigen and any off‐target antigens, cross‐reactivity can be tuned by simply altering the concentration of the soluble TCR.

Another interesting consideration for the specificity of bispecific T‐cell engagers that use anti‐CD3 moieties to redirect T‐cell activation is whether the regulation and thresholds for signal initiation are different from the signal generated by native TCR‐pHLA engagement. Although this has not yet been fully answered, it is an important question because the native TCR is regulated by a sophisticated and intricate signalling network that includes the CD3 signalling complex, co‐receptors (e.g. CD8 and CD4) and both co‐stimulatory (e.g. CD28) and co‐inhibitory molecules (e.g. PD‐1). Indeed, it has been shown that T‐cell receptor fusion constructs (TRuCs) that plug into the native TCR‐CD3 signalling complex are more sensitive to antigen, and are also more tightly regulated, compared with CARs that use artificial signalling domains [83]. Thus, the nature by which anti‐CD3‐based bispecific T‐cell engagers initiate the T‐cell signalling machinery could impact the signal generated between on‐target and off‐target TCR binding, potentially altering the therapeutic window compared with the native TCR‐pHLA interaction.

When considering off‐target reactivity of soluble bispecific TCR reagents, it is important to characterise any changes in the specificity profile, including new potential reactivities [39] and test functional cross‐reactivity on appropriate healthy cell lines and critical tissues [84]. Because off‐target reactivity is very likely to be peptide‐specific, *in vivo* testing in animal models is very challenging as even an HLA‐transgenic mouse will present a distinct peptide repertoire compared with human tissue. Thus, specific *in vitro* assays to detect/monitor unwanted reactivity are a critical aspect of engineering TCRs for use as therapeutics, as exemplified by fatal toxicity reported in an adoptive cell therapy trial [40, 42]. In our experience, it is generally advantageous to start with a wild‐type TCR that has a favourable specificity profile (assessed as described in Ref. [39, 84, 85]). For instance, we have demonstrated that TCRs with energetically balanced interactions with multiple peptide side chains show greater peptide selectivity [39] and that engineering wild‐type TCRs with these attributes often results in conservation of these favourable binding characteristics during the affinity engineering process [70]. In contrast, we and others have demonstrated that TCRs that bind with energetic hotspots are prone to high levels of cross‐reactivity and are often associated with autoreactivity [15, 86, 87, 88, 89]. Thus, structural analysis of a candidate TCR in complex with target pHLA is extremely informative for this selection criteria. Recent studies have also explored the idea of rationally engineering a TCR to have a defined specificity profile using structure‐guided approaches (as reviewed in Ref. [90, 91]). Indeed, Smith and colleagues demonstrated that it is possible to change the specificity of a TCR from one antigen to another using a directed evolution approach by altering the binding geometry of the TCR for the new antigen [81, 92]. Further attempts to fine‐tune the specificity of affinity‐enhanced TCRs through computational design have also been reported in which ‘negative’ mutations that disrupt/remove contacts with the HLA surface or mutations that improved the shape complementarity and energetic compatibility of the TCR have been shown to enhance specificity towards a given peptide [93].

### Summary

Although engineering TCR specificity is only just emerging as a practical concept, recent reports have demonstrated proof‐of‐concept feasibility. The evidence suggests that mutations that constrain the TCR towards recognition of a limited set of peptides, either by removing HLA contacts to make the TCR more ‘peptide centric’, or by optimising contacts with multiple peptide side chains to create a more unique binding interface, seem advantageous. Finally, because of the development of more sophisticated means of assessing TCR cross‐reactivity (combinatorial library screens, pHLA display libraries, etc.), it is now becoming easier to test the effects of protein engineering modifications to fine‐tune TCR specificity. Thus, it is likely that *in silico* methods combined with protein display and more sophisticated means of cross‐reactivity screening will continue to emerge as important tools for developing TCR‐based therapeutics in the future.

## Protein engineering challenges for unconventional T‐cell ligands

One key limitation of targeting classical HLA molecules is their polymorphic nature. Therefore, drugs that target these molecules will always carry some level of patient restriction depending on the genetic background of the individual. However, there are several unconventional HLA‐like molecules that are monomorphic, thereby offering the possibility of pan‐population therapies. These include (but are not limited to) HLA‐E, MHC‐related protein 1 (MR1) and cluster of differentiation 1 a, b, c and d (CD1a, CD1b, CD1c or CD1d), all of which have a very similar overall structure to classical HLA class I. Interestingly, despite their very different chemical nature, TCRs have been shown to interact with these ligands with similar affinities (in the micromolar range) and binding modes, compared with classical TCR‐pHLA interactions [94]. However, major protein engineering challenges exist for the therapeutic targeting of these unconventional HLA‐like molecules, including their stability as soluble proteins, identification of disease‐specific ligands and understanding how to engineer TCRs with enhanced affinity and desirable selectivity. Some of these considerations are discussed below for HLA‐E, MR1 and the CD1 family.

HLA‐E is a class Ib HLA with limited polymorphism [95, 96] expressed by nearly every nucleated cell in the body [97, 98]. Expression of HLA‐E on the cell surface (Fig. 1) is usually reliant on binding of leader peptides from HLA‐A, HLA‐B, HLA‐C or HLA‐G molecules, typically with the sequence VMAPRTL(L/V/I)L [99]. Emerging evidence indicates that HLA‐E is also involved in T‐cell pathogen surveillance, with HLA‐E‐restricted peptides derived from HIV, HBV and tuberculosis implicated as ligands for protective T‐cell‐mediated immunity [100, 101, 102]. Given its broad expression profile, restricted polymorphism and immunogenic capacity, HLA‐E is an attractive therapeutic target. However, a major protein engineering challenge of HLA‐E targeting TCRs is tuning their specificity so that they do not cross‐react with leader peptides present on the surface of virtually all nucleated cells. Additionally, some of the pathogenic peptides identified so far have been shown to generate unstable HLA‐E molecules [102], making their use for the selection and affinity enhancement of TCRs extremely challenging. Consequently, multiple approaches, including the introduction of artificial disulfides that bridge across the binding groove, or that covalently link the peptide to the heavy chain, have been employed to stabilise pHLA‐E [103, 104]. Whether these approaches will prove effective for the selection and engineering of therapeutic HLA‐E targeting TCR molecules remains to be proven.

MR1 also has limited polymorphism [105, 106] and is expressed on virtually every nucleated cell in the body [107]. However, unlike HLA‐E and classical HLA class I, MR1 has been shown to present small chemical compounds (Fig. 1), of which the best characterised is the microbially derived 5‐(2‐oxopropylideneamino)‐6‐d‐ribitylaminouracil (5‐OP‐RU), a short‐lived derivative in the riboflavin pathway [108]. Recent evidence has demonstrated that MR1 can also present other small molecule ligands, including drugs [109], nonmicrobial MR1 ligands [110, 111] and even undefined tumour‐associated ligands [112]. Therefore, like HLA‐E, MR1 is a highly attractive and exciting future therapeutic target. The main protein engineering challenges for targeting MR1 are in identifying the disease‐associated ligands and generating these complexes as soluble molecules for the selection and characterisation of engineered TCRs. It is also unclear whether the nature of the small molecule antigens presented by MR1 will allow the engineering of affinity‐enhanced TCRs with the desired ligand specificity, which will be extremely important due to the ubiquitous expression of MR1 in human tissues.

Unlike HLA‐E, MR1 and classical HLA class I molecules, the monomorphic CD1 molecules present lipid antigens (Fig. 1) and are more limited in their tissue expression. For example, CD1c is only expressed on cells of haematopoietic origin, such as thymocytes and mature haematopoietic cells (e.g. B lymphocytes and myeloid/monocytic antigen‐presenting cells). Although this limits them as targets for many diseases, they are ideally suited for targeting tissue‐specific conditions, where restricted expression could limit the possibility of toxicity in other healthy tissues. However, isolating, characterising and refolding CD1 molecules with disease selective lipids is challenging due to the hydrophobic nature of the ligands, and the fact that the lipid antigens bind deep within the groove of the various CD1 molecules, making them difficult to exchange and extract. Furthermore, a major question remains concerning the nature of TCR recognition of CD1 complexes, particularly whether TCRs can bind independently of the lipid, to a family of related lipids or to a single lipid [113]. A better understanding of this binding mode is essential for engineering CD1‐targeting TCR‐based reagents with the desired/expected specificity.

In summary, the TCR stabilisation approaches discussed herein would likely be transferable to TCRs targeting the unconventional HLA‐like molecules. However, engineering these TCRs for enhanced affinity involves a number of protein engineering challenges, including generation of stable unconventional HLA‐like molecules presenting disease‐relevant ligands for the affinity selection of engineered TCRs, as well as approaches that ensure the fidelity of these engineered TCRs against the intended target. Structure‐guided approaches and learnings from efforts to generate specific affinity‐enhanced soluble TCR for classical pHLA class I molecules are likely to be highly valuable to solve these challenges.

## Concluding remarks

The TCR has obvious therapeutic targeting potential *via* its natural ability to interrogate the proteomic (*via* HLA presentation), metabolomic (*via* MR1 presentation) and lipidomic (*via* CD1 presentation) status of cells to detect aberrant or dysregulated signals. However, due to its poor natural stability as a soluble protein and its relatively weak affinity, its usefulness as a soluble drug is limited. To overcome this issue, several protein engineering efforts have been developed, including the introduction of mutations that stabilise the soluble TCR, and the development of library selections and *in silico* efforts to increase the affinity of the receptor. More bespoke stabilisation of TCRs using unbiased display methods and computational screening has highlighted the potential to further optimise the developability properties of individual TCRs. Although a number of techniques have been established to select mutations to enhance the affinity of TCRs towards a target pHLA, it is clear that advances in our understanding of the molecular rules that govern TCR‐pHLA specificity, as well as structure‐guided approaches, offer new opportunities to further optimise both the affinity and specificity of TCRs developed for therapeutic applications. Finally, it will be interesting to apply these engineering approaches to other TCR ligands, with a view to generate soluble TCR‐based therapeutics that can bypass HLA restriction.

## Conflict of interest

RAR, CM, MLM and DKC are employees of Immunocore Ltd.

## Author contributions

RAR, CM, MLM and DKC all contributed to the writing of this review article.

## References

[1] Garrido F , Cabrera T & Aptsiauri N (2010) "Hard" and "soft" lesions underlying the HLA class I alterations in cancer cells: implications for immunotherapy. Int J Cancer 127, 249–256. 20178101 10.1002/ijc.25270

[2] Berah M , Hors J & Dausset J (1970) A study of HL‐A antigens in human organs. Transplantation 9, 185–192. 5437618 10.1097/00007890-197003000-00001

[3] Williams KA , Hart DN , Fabre JW & Morris PJ (1980) Distribution and quantitation of HLA‐ABC and DR (Ia) antigens on human kidney and other tissues. Transplantation 29, 274–279. 6989046 10.1097/00007890-198004000-00002

[4] Bjorkman PJ , Saper MA , Samraoui B , Bennett WS , Strominger JL & Wiley DC (1987) Structure of the human class I histocompatibility antigen, HLA‐A2. Nature 329, 506–512. 3309677 10.1038/329506a0

[5] Garboczi DN , Ghosh P , Utz U , Fan QR , Biddison WE & Wiley DC (1996) Structure of the complex between human T‐cell receptor, viral peptide and HLA‐A2. Nature 384, 134–141. 8906788 10.1038/384134a0

[6] Garcia KC , Degano M , Stanfield RL , Brunmark A , Jackson MR , Peterson PA , Teyton L & Wilson IA (1996) An alphabeta T cell receptor structure at 2.5 A and its orientation in the TCR‐MHC complex. Science 274, 209–219. 8824178 10.1126/science.274.5285.209

[7] Rossjohn J , Gras S , Miles JJ , Turner SJ , Godfrey DI & McCluskey J (2015) T cell antigen receptor recognition of antigen‐presenting molecules. Annu Rev Immunol 33, 169–200. 25493333 10.1146/annurev-immunol-032414-112334

[8] Rudolph MG , Stanfield RL & Wilson IA (2006) How TCRs bind MHCs, peptides, and coreceptors. Annu Rev Immunol 24, 419–466. 16551255 10.1146/annurev.immunol.23.021704.115658

[9] Liddy N , Bossi G , Adams KJ , Lissina A , Mahon TM , Hassan NJ , Gavarret J , Bianchi FC , Pumphrey NJ , Ladell K *et al*. (2012) Monoclonal TCR‐redirected tumor cell killing. Nat Med 18, 980–987. 22561687 10.1038/nm.2764

[10] Lowe KL , Cole D , Kenefeck R , OKelly I , Lepore M & Jakobsen BK (2019) Novel TCR‐based biologics: mobilising T cells to warm 'cold' tumours. Cancer Treat Rev 77, 35–43. 31207478 10.1016/j.ctrv.2019.06.001

[11] Boulter JM , Glick M , Todorov PT , Baston E , Sami M , Rizkallah P & Jakobsen BK (2003) Stable, soluble T‐cell receptor molecules for crystallization and therapeutics. Protein Eng 16, 707–711. 14560057 10.1093/protein/gzg087

[12] Bridgeman JS , Sewell AK , Miles JJ , Price DA & Cole DK (2012) Structural and biophysical determinants of alphabeta T‐cell antigen recognition. Immunology 135, 9–18. 22044041 10.1111/j.1365-2567.2011.03515.x PMC3246648

[13] Aleksic M , Liddy N , Molloy PE , Pumphrey N , Vuidepot A , Chang KM & Jakobsen BK (2012) Different affinity windows for virus and cancer‐specific T‐cell receptors: implications for therapeutic strategies. Eur J Immunol 42, 3174–3179. 22949370 10.1002/eji.201242606 PMC3776049

[14] Cole DK , Pumphrey NJ , Boulter JM , Sami M , Bell JI , Gostick E , Price DA , Gao GF , Sewell AK & Jakobsen BK (2007) Human TCR‐binding affinity is governed by MHC class restriction. J Immunol 178, 5727–5734. 17442956 10.4049/jimmunol.178.9.5727

[15] Cole DK , van den Berg HA , Lloyd A , Crowther MD , Beck K , Ekeruche‐Makinde J , Miles JJ , Bulek AM , Dolton G , Schauenburg AJ *et al*. (2017) Structural Mechanism Underpinning Cross‐reactivity of a CD8+ T‐cell Clone That Recognizes a Peptide Derived from Human Telomerase Reverse Transcriptase. J Biol Chem. 292, 802–813. 27903649 10.1074/jbc.M116.741603 PMC5247654

[16] Mason D (1998) A very high level of crossreactivity is an essential feature of the T‐cell receptor. Immunol Today 19, 395–404. 9745202 10.1016/s0167-5699(98)01299-7

[17] Sewell AK (2012) Why must T cells be cross‐reactive? Nat Rev Immunol 12, 669–677. 22918468 10.1038/nri3279 PMC7097784

[18] Wooldridge L , Ekeruche‐Makinde J , van den Berg HA , Skowera A , Miles JJ , Tan MP , Dolton G , Clement M , Llewellyn‐Lacey S , Price DA *et al*. (2012) A single autoimmune T cell receptor recognizes more than a million different peptides. J Biol Chem 287, 1168–1177. 22102287 10.1074/jbc.M111.289488 PMC3256900

[19] Schmid DA , Irving MB , Posevitz V , Hebeisen M , Posevitz‐Fejfar A , Sarria JC , Gomez‐Eerland R , Thome M , Schumacher TN , Romero P *et al*. (2010) Evidence for a TCR affinity threshold delimiting maximal CD8 T cell function. J Immunol 184, 4936–4946. 20351194 10.4049/jimmunol.1000173

[20] Tan MP , Gerry AB , Brewer JE , Melchiori L , Bridgeman JS , Bennett AD , Pumphrey NJ , Jakobsen BK , Price DA , Ladell K & *et al*. (2015) T cell receptor binding affinity governs the functional profile of cancer‐specific CD8+ T cells. Clin Exp Immunol 180, 255–270. 25496365 10.1111/cei.12570 PMC4408161

[21] Valitutti S , Muller S , Cella M , Padovan E & Lanzavecchia A (1995) Serial triggering of many T‐cell receptors by a few peptide‐MHC complexes. Nature 375, 148–151. 7753171 10.1038/375148a0

[22] Lever M , Maini PK , van der Merwe PA & Dushek O (2014) Phenotypic models of T cell activation. Nat Rev Immunol 14, 619–629. 25145757 10.1038/nri3728

[23] Moon EK , Wang LC , Dolfi DV , Wilson CB , Ranganathan R , Sun J , Kapoor V , Scholler J , Pure E , Milone MC *et al*. (2014) Multifactorial T‐cell hypofunction that is reversible can limit the efficacy of chimeric antigen receptor‐transduced human T cells in solid tumors. Clin Cancer Res 20, 4262–4273. 24919573 10.1158/1078-0432.CCR-13-2627 PMC4134701

[24] Michaeli Y , Denkberg G , Sinik K , Lantzy L , Chih‐Sheng C , Beauverd C , Ziv T , Romero P & Reiter Y (2009) Expression hierarchy of T cell epitopes from melanoma differentiation antigens: unexpected high level presentation of tyrosinase‐HLA‐A2 Complexes revealed by peptide‐specific. MHC‐restricted, TCR‐like antibodies, J Immunol 182, 6328–6341. 19414786 10.4049/jimmunol.0801898

[25] Li Y , Moysey R , Molloy PE , Vuidepot AL , Mahon T , Baston E , Dunn S , Liddy N , Jacob J , Jakobsen BK & *et al*. (2005) Directed evolution of human T‐cell receptors with picomolar affinities by phage display. Nat Biotechnol 23, 349–354. 15723046 10.1038/nbt1070

[26] Biddison WE , Turner RV , Gagnon SJ , Lev A , Cohen CJ & Reiter Y (2003) Tax and M1 peptide/HLA‐A2‐specific Fabs and T cell receptors recognize nonidentical structural features on peptide/HLA‐A2 complexes. J Immunol 171, 3064–3074. 12960332 10.4049/jimmunol.171.6.3064

[27] Chang AY , Dao T , Gejman RS , Jarvis CA , Scott A , Dubrovsky L , Mathias MD , Korontsvit T , Zakhaleva V , Curcio M *et al*. (2017) A therapeutic T cell receptor mimic antibody targets tumor‐associated PRAME peptide/HLA‐I antigens. J Clin Invest 127, 3557. 10.1172/JCI96860 PMC5669580 28862643

[28] Held G , Wadle A , Dauth N , Stewart‐Jones G , Sturm C , Thiel M , Zwick C , Dieckmann D , Schuler G , Hoogenboom HR *et al*. (2007) MHC‐peptide‐specific antibodies reveal inefficient presentation of an HLA‐A*0201‐restricted. Melan‐A‐derived peptide after active intracellular processing, Eur J Immunol 37, 2008–2017. 10.1002/eji.200636545 17559180

[29] Sastry KS , Too CT , Kaur K , Gehring AJ , Low L , Javiad A , Pollicino T , Li L , Kennedy PT , Lopatin U *et al*. (2011) Targeting hepatitis B virus‐infected cells with a T‐cell receptor‐like antibody. J Virol 85, 1935–1942. 21159876 10.1128/JVI.01990-10 PMC3067764

[30] Yamano Y , Cohen CJ , Takenouchi N , Yao K , Tomaru U , Li HC , Reiter Y & Jacobson S (2004) Increased expression of human T lymphocyte virus type I (HTLV‐I) Tax11‐19 peptide‐human histocompatibility leukocyte antigen A*201 complexes on CD4+ CD25+ T Cells detected by peptide‐specific, major histocompatibility complex‐restricted antibodies in patients with HTLV‐I‐associated neurologic disease. J Exp Med 199, 1367–1377. 15136590 10.1084/jem.20032042 PMC2211812

[31] Ataie N , Xiang J , Cheng N , Brea EJ , Lu W , Scheinberg DA , Liu C & Ng HL (2016) Structure of a TCR‐Mimic Antibody with Target Predicts Pharmacogenetics. J Mol Biol 428, 194–205. 26688548 10.1016/j.jmb.2015.12.002 PMC4738012

[32] Dao T , Pankov D , Scott A , Korontsvit T , Zakhaleva V , Xu Y , Xiang J , Yan S , de Morais Guerreiro MD , Veomett N *et al*. (2015) Therapeutic bispecific T‐cell engager antibody targeting the intracellular oncoprotein WT1. Nat Biotechnol 33, 1079–1086. 26389576 10.1038/nbt.3349 PMC4600043

[33] Maus MV , Plotkin J , Jakka G , Stewart‐Jones G , Riviere I , Merghoub T , Wolchok J , Renner C & Sadelain M (2016) An MHC‐restricted antibody‐based chimeric antigen receptor requires TCR‐like affinity to maintain antigen specificity. Mol Ther Oncolytics 3, 1–9. 29675462 10.1038/mto.2016.23 PMC5904357

[34] Stewart‐Jones G , Wadle A , Hombach A , Shenderov E , Held G , Fischer E , Kleber S , Nuber N , Stenner‐Liewen F , Bauer S *et al*. (2009) Rational development of high‐affinity T‐cell receptor‐like antibodies. Proc Natl Acad Sci U S A 106, 5784–5788. 19307587 10.1073/pnas.0901425106 PMC2667008

[35] Zhao Q , Ahmed M , Tassev DV , Hasan A , Kuo TY , Guo HF , O'Reilly RJ & Cheung NK (2015) Affinity maturation of T‐cell receptor‐like antibodies for Wilms tumor 1 peptide greatly enhances therapeutic potential. Leukemia 29, 2238–2247. 25987253 10.1038/leu.2015.125 PMC4788467

[36] Chames P , Willemsen RA , Rojas G , Dieckmann D , Rem L , Schuler G , Bolhuis RL & Hoogenboom HR (2002) TCR‐like human antibodies expressed on human CTLs mediate antibody affinity‐dependent cytolytic activity. J Immunol 169, 1110–1118. 12097420 10.4049/jimmunol.169.2.1110

[37] Hulsmeyer M , Chames P , Hillig RC , Stanfield RL , Held G , Coulie PG , Alings C , Wille G , Saenger W , Uchanska‐Ziegler B *et al*. (2005) A major histocompatibility complex‐peptide‐restricted antibody and t cell receptor molecules recognize their target by distinct binding modes: crystal structure of human leukocyte antigen (HLA)‐A1‐MAGE‐A1 in complex with FAB‐HYB3. J Biol Chem 280, 2972–2980. 15537658 10.1074/jbc.M411323200

[38] Oren R , Hod‐Marco M , Haus‐Cohen M , Thomas S , Blat D , Duvshani N , Denkberg G , Elbaz Y , Benchetrit F , Eshhar Z *et al*. (2014) Functional comparison of engineered T cells carrying a native TCR versus TCR‐like antibody‐based chimeric antigen receptors indicates affinity/avidity thresholds. J Immunol 193, 5733–5743. 25362181 10.4049/jimmunol.1301769

[39] Holland CJ , Crean RM , Pentier JM , de Wet B , Lloyd A , Srikannathasan V , Lissin N , Lloyd KA , Blicher TH , Conroy PJ *et al*. (2020) Specificity of bispecific T cell receptors and antibodies targeting peptide‐HLA. J Clin Invest 130, 2673–2688. 32310221 10.1172/JCI130562 PMC7190993

[40] Cameron BJ , Gerry AB , Dukes J , Harper JV , Kannan V , Bianchi FC , Grand F , Brewer JE , Gupta M , Plesa G *et al*. (2013) Identification of a Titin‐derived HLA‐A1‐presented peptide as a cross‐reactive target for engineered MAGE A3‐directed T cells. Sci Transl Med 5, 197ra103. 10.1126/scitranslmed.3006034 PMC6002776 23926201

[41] Linette GP , Stadtmauer EA , Maus MV , Rapoport AP , Levine BL , Emery L , Litzky L , Bagg A , Carreno BM , Cimino PJ *et al*. (2013) Cardiovascular toxicity and titin cross‐reactivity of affinity‐enhanced T cells in myeloma and melanoma. Blood 122, 863–871. 23770775 10.1182/blood-2013-03-490565 PMC3743463

[42] Raman MC , Rizkallah PJ , Simmons R , Donnellan Z , Dukes J , Bossi G , Le Provost GS , Todorov P , Baston E , Hickman E *et al*. (2016) Direct molecular mimicry enables off‐target cardiovascular toxicity by an enhanced affinity TCR designed for cancer immunotherapy. Sci Rep 6, 18851. 26758806 10.1038/srep18851 PMC4725365

[43] Heemskerk MH , Hagedoorn RS , van der Hoorn MA , van der Veken LT , Hoogeboom M , Kester MG , Willemze R & Falkenburg JH (2007) Efficiency of T‐cell receptor expression in dual‐specific T cells is controlled by the intrinsic qualities of the TCR chains within the TCR‐CD3 complex. Blood 109, 235–243. 16968899 10.1182/blood-2006-03-013318

[44] Howie B , Sherwood AM , Berkebile AD , Berka J , Emerson RO , Williamson DW , Kirsch I , Vignali M , Rieder MJ , Carlson CS & *et al*. (2015) High‐throughput pairing of T cell receptor alpha and beta sequences. Sci Transl Med 7, 301ra131. 10.1126/scitranslmed.aac5624 26290413

[45] Shcherbinin DS , Belousov VA & Shugay M (2020) Comprehensive analysis of structural and sequencing data reveals almost unconstrained chain pairing in TCRalphabeta complex. PLoS Comput Biol 16, e1007714. 32163410 10.1371/journal.pcbi.1007714 PMC7093030

[46] Carter JA , Preall JB , Grigaityte K , Goldfless SJ , Jeffery E , Briggs AW , Vigneault F & Atwal GS (2019) Single T Cell Sequencing Demonstrates the Functional Role of αβ TCR Pairing in Cell Lineage and Antigen Specificity. Front Immunol 10. 10.3389/fimmu.2019.01516 PMC6684766 31417541

[47] Novotny J , Ganju RK , Smiley ST , Hussey RE , Luther MA , Recny MA , Siliciano RF & Reinherz EL (1991) A soluble, single‐chain T‐cell receptor fragment endowed with antigen‐combining properties. Proc Natl Acad Sci U S A 88, 8646–8650. 1924326 10.1073/pnas.88.19.8646 PMC52566

[48] Hoo WF , Lacy MJ , Denzin LK , Voss EW Jr , Hardman KD & Kranz DM (1992) Characterization of a single‐chain T‐cell receptor expressed in Escherichia coli. Proc Natl Acad Sci U S A 89, 4759–4763. 1584815 10.1073/pnas.89.10.4759 PMC49163

[49] Richman SA , Aggen DH , Dossett ML , Donermeyer DL , Allen PM , Greenberg PD & Kranz DM (2009) Structural features of T cell receptor variable regions that enhance domain stability and enable expression as single‐chain ValphaVbeta fragments. Mol Immunol 46, 902–916. 18962897 10.1016/j.molimm.2008.09.021 PMC2666936

[50] Gunnarsen KS , Kristinsson SG , Justesen S , Frigstad T , Buus S , Bogen B , Sandlie I & Loset GA (2013) Chaperone‐assisted thermostability engineering of a soluble T cell receptor using phage display. Sci Rep 3, 1162. 23362461 10.1038/srep01162 PMC3557450

[51] Gregoire C , Rebai N , Schweisguth F , Necker A , Mazza G , Auphan N , Millward A , Schmitt‐Verhulst AM & Malissen B (1991) Engineered secreted T‐cell receptor alpha beta heterodimers. Proc Natl Acad Sci U S A 88, 8077–8081. 1716770 10.1073/pnas.88.18.8077 PMC52449

[52] Chang HC , Bao Z , Yao Y , Tse AG , Goyarts EC , Madsen M , Kawasaki E , Brauer PP , Sacchettini JC , Nathenson SG *et al*. (1994) A general method for facilitating heterodimeric pairing between two proteins: application to expression of alpha and beta T‐cell receptor extracellular segments. Proc Natl Acad Sci U S A 91, 11408–11412. 7972074 10.1073/pnas.91.24.11408 PMC45240

[53] Willcox BE , Gao GF , Wyer JR , O'Callaghan CA , Boulter JM , Jones EY , van der Merwe PA , Bell JI & Jakobsen BK (1999) Production of soluble alphabeta T‐cell receptor heterodimers suitable for biophysical analysis of ligand binding. Protein science : a publication of the Protein Society 8, 2418–2423. 10595544 10.1110/ps.8.11.2418 PMC2144200

[54] van Boxel GI , Stewart‐Jones G , Holmes S , Sainsbury S , Shepherd D , Gillespie GM , Harlos K , Stuart DI , Owens R & Jones EY (2009) Some lessons from the systematic production and structural analysis of soluble (alpha)(beta) T‐cell receptors. J Immunol Methods 350, 14–21. 19715696 10.1016/j.jim.2009.08.008

[55] Boulter JM & Jakobsen BK (2005) Stable, soluble, high‐affinity, engineered T cell receptors: novel antibody‐like proteins for specific targeting of peptide antigens. Clin Exp Immunol 142, 454–460. 16297157 10.1111/j.1365-2249.2005.02929.x PMC1809535

[56] Thomas S , Mohammed F , Reijmers RM , Woolston A , Stauss T , Kennedy A , Stirling D , Holler A , Green L , Jones D *et al*. (2019) Framework engineering to produce dominant T cell receptors with enhanced antigen‐specific function. Nat Commun 10, 4451. 31575864 10.1038/s41467-019-12441-w PMC6773850

[57] Froning K , Maguire J , Sereno A , Huang F , Chang S , Weichert K , Frommelt AJ , Dong J , Wu X , Austin H *et al*. (2020) Computational stabilization of T cell receptors allows pairing with antibodies to form bispecifics. Nat Commun 11, 2330. 32393818 10.1038/s41467-020-16231-7 PMC7214467

[58] Sadio F , Stadlmayr G , Stadlbauer K , Graf M , Scharrer A , Ruker F & Wozniak‐Knopp G (2020) Stabilization of soluble high‐affinity T‐cell receptor with de novo disulfide bonds. FEBS Lett 594, 477–490. 31552676 10.1002/1873-3468.13616 PMC7027902

[59] Kieke MC , Shusta EV , Boder ET , Teyton L , Wittrup KD & Kranz DM (1999) Selection of functional T cell receptor mutants from a yeast surface‐display library. Proc Natl Acad Sci U S A 96, 5651–5656. 10318939 10.1073/pnas.96.10.5651 PMC21915

[60] Sharma P & Kranz DM (2018) Subtle changes at the variable domain interface of the T‐cell receptor can strongly increase affinity. J Biol Chem 293, 1820–1834. 29229779 10.1074/jbc.M117.814152 PMC5798310

[61] Koenig P , Lee CV , Walters BT , Janakiraman V , Stinson J , Patapoff TW & Fuh G (2017) Mutational landscape of antibody variable domains reveals a switch modulating the interdomain conformational dynamics and antigen binding. Proc Natl Acad Sci U S A 114, E486–E495. 28057863 10.1073/pnas.1613231114 PMC5278476

[62] Harris DT , Wang N , Riley TP , Anderson SD , Singh NK , Procko E , Baker BM & Kranz DM (2016) Deep Mutational Scans as a Guide to Engineering High Affinity T Cell Receptor Interactions with Peptide‐bound Major Histocompatibility Complex. J Biol Chem 291, 24566–24578. 27681597 10.1074/jbc.M116.748681 PMC5114409

[63] Jain T , Sun T , Durand S , Hall A , Houston NR , Nett JH , Sharkey B , Bobrowicz B , Caffry I , Yu Y *et al*. (2017) Biophysical properties of the clinical‐stage antibody landscape. Proc Natl Acad Sci U S A 114, 944–949. 28096333 10.1073/pnas.1616408114 PMC5293111

[64] Raybould MIJ , Marks C , Krawczyk K , Taddese B , Nowak J , Lewis AP , Bujotzek A , Shi J & Deane CM (2019) Five computational developability guidelines for therapeutic antibody profiling. Proc Natl Acad Sci U S A 116, 4025–4030. 30765520 10.1073/pnas.1810576116 PMC6410772

[65] Haidar JN , Pierce B , Yu Y , Tong W , Li M & Weng Z (2009) Structure‐based design of a T‐cell receptor leads to nearly 100‐fold improvement in binding affinity for pepMHC. Proteins 74, 948–960. 18767161 10.1002/prot.22203 PMC2696811

[66] Irving M , Zoete V , Hebeisen M , Schmid D , Baumgartner P , Guillaume P , Romero P , Speiser D , Luescher I , Rufer N & *et al*. (2012) Interplay between T cell receptor binding kinetics and the level of cognate peptide presented by major histocompatibility complexes governs CD8+ T cell responsiveness. J Biol Chem 287, 23068–23078. 22549784 10.1074/jbc.M112.357673 PMC3391157

[67] Pierce BG , Hellman LM , Hossain M , Singh NK , Vander Kooi CW , Weng Z & Baker BM (2014) Computational design of the affinity and specificity of a therapeutic T cell receptor. PLoS Comput Biol 10, e1003478. 24550723 10.1371/journal.pcbi.1003478 PMC3923660

[68] Zoete V , Irving M , Ferber M , Cuendet MA & Michielin O (2013) Structure‐Based, Rational Design of T Cell Receptors. Front Immunol 4, 268. 24062738 10.3389/fimmu.2013.00268 PMC3770923

[69] Malecek K , Grigoryan A , Zhong S , Gu WJ , Johnson LA , Rosenberg SA , Cardozo T & Krogsgaard M (2014) Specific increase in potency via structure‐based design of a TCR. J Immunol 193, 2587–2599. 25070852 10.4049/jimmunol.1302344 PMC4205480

[70] Crean RM , MacLachlan BJ , Madura F , Whalley T , Rizkallah PJ , Holland CJ , McMurran C , Harper S , Godkin A , Sewell AK *et al*. (2020) Molecular Rules Underpinning Enhanced Affinity Binding of Human T Cell Receptors Engineered for Immunotherapy. Mol Ther Oncolytics 18, 443–456. 32913893 10.1016/j.omto.2020.07.008 PMC7452143

[71] Chervin AS , Aggen DH , Raseman JM & Kranz DM (2008) Engineering higher affinity T cell receptors using a T cell display system. J Immunol Methods 339, 175–184. 18854190 10.1016/j.jim.2008.09.016 PMC2680719

[72] Wagner EK , Qerqez AN , Stevens CA , Nguyen AW , Delidakis G & Maynard JA (2019) Human cytomegalovirus‐specific T‐cell receptor engineered for high affinity and soluble expression using mammalian cell display. J Biol Chem 294, 5790–5804. 30796163 10.1074/jbc.RA118.007187 PMC6463697

[73] Vazquez‐Lombardi R , Jung JS , Bieberich F , Kapetanovic E , Aznauryan E , Weber CR & Reddy ST . (2020) CRISPR‐targeted display of functional T cell receptors enables engineering of enhanced specificity and prediction of cross‐reactivity. *bioRxiv* [PREPRINT], 2020.06.23.166363.

[74] Kessels HW , van Den Boom MD , Spits H , Hooijberg E & Schumacher TN (2000) Changing T cell specificity by retroviral T cell receptor display. Proc Natl Acad Sci U S A 97, 14578–14583. 11121060 10.1073/pnas.97.26.14578 PMC18961

[75] Malecek K , Zhong S , McGary K , Yu C , Huang K , Johnson LA , Rosenberg SA & Krogsgaard M (2013) Engineering improved T cell receptors using an alanine‐scan guided T cell display selection system. J Immunol Methods 392, 1–11. 23500145 10.1016/j.jim.2013.02.018 PMC3668434

[76] Holler PD , Holman PO , Shusta EV , O'Herrin S , Wittrup KD & Kranz DM (2000) In vitro evolution of a T cell receptor with high affinity for peptide/MHC. Proc Natl Acad Sci U S A 97, 5387–5392. 10779548 10.1073/pnas.080078297 PMC25838

[77] Shusta EV , Holler PD , Kieke MC , Kranz DM & Wittrup KD (2000) Directed evolution of a stable scaffold for T‐cell receptor engineering. Nat Biotechnol 18, 754–759. 10888844 10.1038/77325

[78] Smith SN , Harris DT & Kranz DM (2015) T Cell Receptor Engineering and Analysis Using the Yeast Display Platform. Methods in molecular biology (Clifton, NJ). 1319, 95–141. 10.1007/978-1-4939-2748-7_6 PMC5562502 26060072

[79] Fergusson JR , Wallace Z , Connolly MM , Woon AP , Suckling RJ , Hine DW , Barber C , Bunjobpol W , Choi BS , Crespillo S *et al*. (2020) Immune‐mobilising monoclonal T cell receptors mediate specific and rapid elimination of Hepatitis B‐infected cells, *Hepatology* . Baltimore, Md. 10.1002/hep.31503 PMC7702151 32770836

[80] Clarkson T & Lowman HB (2004) Phage Display: A Practical Approach. Oxford University Press, Oxford.

[81] Harris DT , Singh NK , Cai Q , Smith SN , Vander Kooi C , Procko E , Kranz DM & Baker BM (2016) An Engineered Switch in T Cell Receptor Specificity Leads to an Unusual but Functional Binding Geometry. Structure 24, 1142–1154. 27238970 10.1016/j.str.2016.04.011 PMC4938795

[82] Pettmann J , Abu‐Shah E , Kutuzov M , Wilson DB , Dustin ML , Davis SJ , van der Merwe PA & Dushek O . (2020) T cells exhibit unexpectedly low discriminatory power and can respond to ultra‐low affinity peptide‐MHC ligands. *bioRxiv* [PREPRINT], 2020.11.14.382630.

[83] Baeuerle PA , Ding J , Patel E , Thorausch N , Horton H , Gierut J , Scarfo I , Choudhary R , Kiner O , Krishnamurthy J *et al*. (2019) Synthetic TRuC receptors engaging the complete T cell receptor for potent anti‐tumor response. Nat Commun 10, 2087. 31064990 10.1038/s41467-019-10097-0 PMC6504948

[84] Harper J , Adams KJ , Bossi G , Wright DE , Stacey AR , Bedke N , Martinez‐Hague R , Blat D , Humbert L , Buchanan H *et al*. (2018) An approved in vitro approach to preclinical safety and efficacy evaluation of engineered T cell receptor anti‐CD3 bispecific (ImmTAC) molecules. PLoS One 13, e0205491. 30321203 10.1371/journal.pone.0205491 PMC6188753

[85] Coles CH , Mulvaney RM , Malla S , Walker A , Smith KJ , Lloyd A , Lowe KL , McCully ML , Martinez Hague R , Aleksic M *et al*. (2020) TCRs with Distinct Specificity Profiles Use Different Binding Modes to Engage an Identical Peptide‐HLA Complex. J Immunol 204, 1943–1953. 32102902 10.4049/jimmunol.1900915 PMC7086387

[86] Adams JJ , Narayanan S , Birnbaum ME , Sidhu SS , Blevins SJ , Gee MH , Sibener LV , Baker BM , Kranz DM & Garcia KC (2016) Structural interplay between germline interactions and adaptive recognition determines the bandwidth of TCR‐peptide‐MHC cross‐reactivity. Nat Immunol 17, 87–94. 26523866 10.1038/ni.3310 PMC4684756

[87] Cole DK , Bulek AM , Dolton G , Schauenberg AJ , Szomolay B , Rittase W , Trimby A , Jothikumar P , Fuller A , Skowera A *et al*. (2016) Hotspot autoimmune T cell receptor binding underlies pathogen and insulin peptide cross‐reactivity. J Clin Invest 126, 2191–2204. 27183389 10.1172/JCI85679 PMC4887163

[88] Hahn M , Nicholson MJ , Pyrdol J & Wucherpfennig KW (2005) Unconventional topology of self peptide‐major histocompatibility complex binding by a human autoimmune T cell receptor. Nat Immunol 6, 490–496. 15821740 10.1038/ni1187 PMC3415330

[89] Harkiolaki M , Holmes SL , Svendsen P , Gregersen JW , Jensen LT , McMahon R , Friese MA , van Boxel G , Etzensperger R , Tzartos JS *et al*. (2009) T cell‐mediated autoimmune disease due to low‐affinity crossreactivity to common microbial peptides. Immunity 30, 348–357. 19303388 10.1016/j.immuni.2009.01.009

[90] Riley TP & Baker BM (2018) The intersection of affinity and specificity in the development and optimization of T cell receptor based therapeutics. Semin Cell Dev Biol 84, 30–41. 30449534 10.1016/j.semcdb.2017.10.017 PMC9026737

[91] Spear TT , Evavold BD , Baker BM & Nishimura MI (2019) Understanding TCR affinity, antigen specificity, and cross‐reactivity to improve TCR gene‐modified T cells for cancer immunotherapy. Cancer Immunol Immunother 68, 1881–1889. 31595324 10.1007/s00262-019-02401-0 PMC11028285

[92] Smith SN , Wang Y , Baylon JL , Singh NK , Baker BM , Tajkhorshid E & Kranz DM (2014) Changing the peptide specificity of a human T‐cell receptor by directed evolution. Nat Commun 5, 5223. 25376839 10.1038/ncomms6223 PMC4225554

[93] Hellman LM , Foley KC , Singh NK , Alonso JA , Riley TP , Devlin JR , Ayres CM , Keller GLJ , Zhang Y , Vander Kooi CW *et al*. (2019) Improving T Cell Receptor On‐Target Specificity via Structure‐Guided Design. Mol Ther 27, 300–313. 30617019 10.1016/j.ymthe.2018.12.010 PMC6369632

[94] Bhati M , Cole DK , McCluskey J , Sewell AK & Rossjohn J (2014) The versatility of the alphabeta T‐cell antigen receptor. Protein science : a publication of the Protein Society 23, 260–272. 24375592 10.1002/pro.2412 PMC3945834

[95] Grimsley C & Ober C (1997) Population genetic studies of HLA‐E: evidence for selection. Hum Immunol 52, 33–40. 9021407 10.1016/S0198-8859(96)00241-8

[96] Strong RK , Holmes MA , Li P , Braun L , Lee N & Geraghty DE (2003) HLA‐E allelic variants. Correlating differential expression, peptide affinities, crystal structures, and thermal stabilities. J Biol Chem 278, 5082–5090. 12411439 10.1074/jbc.M208268200

[97] Geraghty DE , Stockschleader M , Ishitani A & Hansen JA (1992) Polymorphism at the HLA‐E locus predates most HLA‐A and ‐B polymorphism. Hum Immunol 33, 174–184. 1618657 10.1016/0198-8859(92)90069-y

[98] Tomasec P , Braud VM , Rickards C , Powell MB , McSharry BP , Gadola S , Cerundolo V , Borysiewicz LK , McMichael AJ & Wilkinson GW (2000) Surface expression of HLA‐E, an inhibitor of natural killer cells, enhanced by human cytomegalovirus gpUL40. Science 287, 1031. 10669413 10.1126/science.287.5455.1031

[99] Braud V , Jones EY & McMichael A (1997) The human major histocompatibility complex class Ib molecule HLA‐E binds signal sequence‐derived peptides with primary anchor residues at positions 2 and 9. Eur J Immunol 27, 1164–1169. 9174606 10.1002/eji.1830270517

[100] Burwitz BJ , Hashiguchi PK , Mansouri M , Meyer C , Gilbride RM , Biswas S , Womack JL , Reed JS , Wu HL , Axthelm MK *et al*. (2020) MHC‐E‐Restricted CD8(+) T Cells Target Hepatitis B Virus‐Infected Human Hepatocytes. J Immunol 204, 2169–2176. 32161099 10.4049/jimmunol.1900795 PMC8109620

[101] Joosten SA , van Meijgaarden KE , van Weeren PC , Kazi F , Geluk A , Savage ND , Drijfhout JW , Flower DR , Hanekom WA , Klein MR & *et al*. (2010) Mycobacterium tuberculosis peptides presented by HLA‐E molecules are targets for human CD8 T‐cells with cytotoxic as well as regulatory activity. PLoS Pathog 6, e1000782. 20195504 10.1371/journal.ppat.1000782 PMC2829052

[102] Walters LC , Harlos K , Brackenridge S , Rozbesky D , Barrett JR , Jain V , Walter TS , O'Callaghan CA , Borrow P , Toebes M *et al*. (2018) Pathogen‐derived HLA‐E bound epitopes reveal broad primary anchor pocket tolerability and conformationally malleable peptide binding. Nat Commun 9, 3137. 30087334 10.1038/s41467-018-05459-z PMC6081459

[103] Hein Z , Uchtenhagen H , Abualrous ET , Saini SK , Janssen L , Van Hateren A , Wiek C , Hanenberg H , Momburg F , Achour A *et al*. (2014) Peptide‐independent stabilization of MHC class I molecules breaches cellular quality control. J Cell Sci 127, 2885–2897. 24806963 10.1242/jcs.145334

[104] Saini SK , Tamhane T , Anjanappa R , Saikia A , Ramskov S , Donia M , Svane IM , Jakobsen SN , Garcia‐Alai M , Zacharias M *et al*. (2019) Empty peptide‐receptive MHC class I molecules for efficient detection of antigen‐specific T cells. Sci Immunol 4. 10.1126/sciimmunol.aau9039 31324690

[105] Huang S , Gilfillan S , Cella M , Miley MJ , Lantz O , Lybarger L , Fremont DH & Hansen TH (2005) Evidence for MR1 antigen presentation to mucosal‐associated invariant T cells. J Biol Chem 280, 21183–21193. 15802267 10.1074/jbc.M501087200

[106] Lopez‐Sagaseta J , Dulberger CL , McFedries A , Cushman M , Saghatelian A & Adams EJ (2013) MAIT recognition of a stimulatory bacterial antigen bound to MR1. J Immunol 191, 5268–5277. 24108697 10.4049/jimmunol.1301958 PMC3819123

[107] Riegert P , Wanner V & Bahram S (1998) Genomics, isoforms, expression, and phylogeny of the MHC class I‐related MR1 gene. J Immunol 161, 4066–4077. 9780177

[108] Kjer‐Nielsen L , Patel O , Corbett AJ , Le Nours J , Meehan B , Liu L , Bhati M , Chen Z , Kostenko L , Reantragoon R *et al*. (2012) MR1 presents microbial vitamin B metabolites to MAIT cells. Nature 491, 717–723. 23051753 10.1038/nature11605

[109] Keller AN , Eckle SB , Xu W , Liu L , Hughes VA , Mak JY , Meehan BS , Pediongco T , Birkinshaw RW , Chen Z *et al*. (2017) Drugs and drug‐like molecules can modulate the function of mucosal‐associated invariant T cells. Nat Immunol 18, 402–411. 28166217 10.1038/ni.3679

[110] Gherardin NA , Keller AN , Woolley RE , Le Nours J , Ritchie DS , Neeson PJ , Birkinshaw RW , Eckle SBG , Waddington JN , Liu L *et al*. (2016) Diversity of T Cells Restricted by the MHC Class I‐Related Molecule MR1 Facilitates Differential Antigen Recognition. Immunity 44, 32–45. 26795251 10.1016/j.immuni.2015.12.005

[111] Lepore M , Kalinichenko A , Calogero S , Kumar P , Paleja B , Schmaler M , Narang V , Zolezzi F , Poidinger M , Mori L & *et al*. (2017) Functionally diverse human T cells recognize non‐microbial antigens presented by MR1. Elife. 6. 10.7554/eLife.24476 PMC5459576 28518056

[112] Crowther MD , Dolton G , Legut M , Caillaud ME , Lloyd A , Attaf M , Galloway SAE , Rius C , Farrell CP , Szomolay B *et al*. (2020) Genome‐wide CRISPR‐Cas9 screening reveals ubiquitous T cell cancer targeting via the monomorphic MHC class I‐related protein MR1. Nat Immunol 21, 178–185. 31959982 10.1038/s41590-019-0578-8 PMC6983325

[113] Cotton RN , Shahine A , Rossjohn J & Moody DB (2018) Lipids hide or step aside for CD1‐autoreactive T cell receptors. Curr Opin Immunol 52, 93–99. 29738961 10.1016/j.coi.2018.04.013 PMC6004262

[114] Beerli RR , Bauer M , Buser RB , Gwerder M , Muntwiler S , Maurer P , Saudan P & Bachmann MF (2008) Isolation of human monoclonal antibodies by mammalian cell display. Proc Natl Acad Sci U S A 105, 14336–14341. 18812621 10.1073/pnas.0805942105 PMC2567231

[115] Benatuil L , Perez JM , Belk J & Hsieh CM (2010) An improved yeast transformation method for the generation of very large human antibody libraries. Protein Eng Des Sel 23, 155–159. 20130105 10.1093/protein/gzq002

[116] Chao G , Lau WL , Hackel BJ , Sazinsky SL , Lippow SM & Wittrup KD (2006) Isolating and engineering human antibodies using yeast surface display. Nat Protoc 1, 755–768. 17406305 10.1038/nprot.2006.94

[117] Gaidukov L , Wroblewska L , Teague B , Nelson T , Zhang X , Liu Y , Jagtap K , Mamo S , Tseng WA , Lowe A *et al*. (2018) A multi‐landing pad DNA integration platform for mammalian cell engineering. Nucleic Acids Res 46, 4072–4086. 29617873 10.1093/nar/gky216 PMC5934685

[118] Ho M & Pastan I (2009) Mammalian cell display for antibody engineering. Methods in molecular biology (Clifton, NJ). 525 (337–52), xiv. 10.1007/978-1-59745-554-1_18 PMC3475965 19252852

